# Risk factors for prolonged respiratory support in late preterm infants: a LASSO-Cox regression analysis

**DOI:** 10.3389/fped.2026.1832233

**Published:** 2026-06-10

**Authors:** Yu Huang, Xiao-Shuang Bao, Na Sun, Kai Li, Cheng-Cheng Huang, Shi-Fai Zhang

**Affiliations:** First Affiliated Hospital of Wannan Medical College, Wuhu, China

**Keywords:** LASSO-Cox regression, late preterm infants, nomogram, respiratory support, SHAP analysis, weaning prediction

## Abstract

**Objective:**

To investigate determinants of respiratory support duration and construct an interpretable weaning prediction model for late preterm infants.

**Methods:**

A retrospective cohort study enrolled 365 late preterm infants (gestational age 34 + 0–36 + 6 weeks). Respiratory support duration (days) was the survival outcome, with successful weaning as the event. After Pearson correlation screening (|r| > 0.75 excluded), LASSO-Cox regression (10-fold cross-validation) selected predictors, followed by multivariable Cox modeling. Discrimination was assessed by C-index and time-dependent AUC at days 3, 5, and 8, with optimism-corrected bootstrap validation. Calibration was evaluated using calibration curves, Brier scores, and decision curve analysis. SHAP analysis quantified feature contributions on the log-hazard scale. Sensitivity analyses verified robustness.

**Results:**

Multiple pregnancy (HR = 1.289, 95% CI: 1.038–1.601, *P* = 0.022), superoxide dismutase (SOD) (HR = 1.014, 95% CI: 1.009–1.019, *P* < 0.001), and albumin-to-globulin ratio (A/G) (HR = 1.130, 95% CI: 1.001–1.275, *P* = 0.049) were risk factors. Nasal continuous positive airway pressure (NCPAP) (HR = 0.703, 95% CI: 0.564–0.878, *P* = 0.002), lymphocyte percentage (LYM_PC) (HR = 0.987, 95% CI: 0.978–0.997, *P* = 0.008), and lactate dehydrogenase (LDH) (HR = 0.991, 95% CI: 0.984–0.998, *P* = 0.013, per 10-unit increase) were protective factors. Pulmonary surfactant showed no association (*P* = 0.652). The model achieved a C-index of 0.677 (optimism-corrected 0.661) and td-AUC of 0.732, 0.769, and 0.782 at days 3, 5, and 8. Calibration was acceptable, DCA showed net benefit at day 5, and SHAP identified SOD, NCPAP, and LYM_PC as primary drivers.

**Conclusion:**

The LASSO-Cox nomogram demonstrated moderate-to-good discrimination. Multiple pregnancy and elevated SOD are warning signs for prolonged oxygen dependency, while early NCPAP can shorten respiratory support. Nomogram visualization with SHAP interpretability provides a transparent basis for individualized weaning assessment.

## Introduction

Late preterm infants (gestational age 34 + 0–36 + 6 weeks) account for more than 70% of all preterm births. Due to immature respiratory system development, they often require respiratory support treatment after birth (1). Prolonged respiratory support duration increases the risk of complications such as bronchopulmonary dysplasia and ventilator-associated lung injury. Accurate prediction of weaning timing is crucial for optimizing clinical management and improving prognosis (2). However, traditional prognostic assessments often rely on single indicators or simple regression analyses, making it difficult to effectively handle multicollinearity and complex interactions in high-dimensional data.

LASSO-Cox regression achieves variable selection through penalty functions, enabling the identification of key prognostic factors and construction of parsimonious yet effective prediction models in high-dimensional survival data (3). This study follows the TRIPOD (Transparent Reporting of a multivariable prediction model for Individual Prognosis Or Diagnosis) statement for prediction model construction and reporting (4) to ensure transparency and reproducibility. This study aims to utilize LASSO-Cox regression to screen influencing factors of respiratory support duration in late preterm infants and construct a nomogram prediction model. To enhance model interpretability, SHAP (SHapley Additive exPlanations) analysis (5) was employed to quantify the contribution of each variable to individual predictions, providing an individualized and interpretable weaning risk assessment tool for the neonatal intensive care unit (NICU).

## Materials and methods

### Study population

A retrospective analysis was conducted on late preterm infants admitted to our NICU from April 2024 to October 2025. Inclusion criteria: (1) Late preterm infants with gestational age 34 + 0–36 + 6 weeks; (2) Received respiratory support within 24 h after birth (including nasal cannula oxygen, NCPAP, or mechanical ventilation); (3) Complete clinical data including maternal perinatal information and infant laboratory indicators. Exclusion criteria: (1) Presence of severe congenital malformations or chromosomal abnormalities; (2) Prenatal diagnosis of intrauterine growth restriction (IUGR) or twin-to-twin transfusion syndrome (TTTS); (3) Birth asphyxia (Apgar score <=3 for more than 5 min); (4) Death or withdrawal of treatment during the study period.

This study was exempt from ethical approval by the Ethics Committee of the First Affiliated Hospital of Wannan Medical College (Supporting Document 1), and informed consent was waived for retrospective studies.

### Data collection

General Characteristics: Sex (SEX), Gestational Age (GA, weeks), Birth Weight (BW, g), Multiple Pregnancy (MP, twins or higher-order=1, singleton=0), Mode of Delivery (MOD), Placental Abruption (PA), Premature Rupture of Membranes (PROM), Meconium-Stained Amniotic Fluid (MSAF), 1-minute/5-minute/10-minute Apgar Scores;

Respiratory Support: Respiratory Support Outcome (STATUS, successful weaning=1, censored=0), Duration of Respiratory Support (DRS, days), Pulmonary Surfactant Administration (PSA, administered=1, not administered=0), Nasal Cannula Oxygen Therapy (NC), Nasal Continuous Positive Airway Pressure (NCPAP, used=1, not used=0), Synchronized Intermittent Mandatory Ventilation (SIMV);

Maternal Perinatal Indicators: Maternal Gamma-Glutamyl Transferase (M-GGT, U/L), Maternal Alkaline Phosphatase (M-ALP, U/L), Maternal Lactate Dehydrogenase (M-LDH, U/L);

Neonatal Laboratory Parameters:

Blood Gas Analysis: pH, Partial Pressure of Carbon Dioxide (PCO₂, mmHg), Partial Pressure of Oxygen (PO₂, mmHg), Bicarbonate (HCO₃, mmol/L), Lactate (Lac, mmol/L);Complete Blood Count: White Blood Cell Count (WBC,   ×  10⁹/L), Neutrophil Percentage (NEUT%, %), Lymphocyte Percentage (LYM%, %), Red Cell Distribution Width (RDW, %), Platelet Distribution Width (PDW), Hemoglobin (Hb, g/L), Hematocrit (HCT, %), Platelet Count (PLT,   ×  10⁹/L);Coagulation Profile: International Normalized Ratio (INR), Fibrinogen (Fib, g/L), D-Dimer (D-D, mg/L);Myocardial Enzymes: Creatine Kinase-MB (CK-MB, U/L), Lactate Dehydrogenase (LDH, U/L), Hydroxybutyrate Dehydrogenase (HBDH, U/L);Biochemical Markers: Gamma-Glutamyl Transferase (GGT, U/L), Alkaline Phosphatase (ALP, U/L), Albumin-to-Globulin Ratio (A/G ratio), High-Sensitivity C-Reactive Protein (hs-CRP, mg/L), Procalcitonin (PCT, ng/mL), Superoxide Dismutase (SOD, U/mL).

### Outcome definition

Primary outcome: Respiratory support duration, defined as the time interval (days) from first respiratory support after birth to successful weaning (cessation of oxygen with stable respiration for >=24 h). Censoring definition: Transfer or death without weaning during hospitalization, or no weaning within the study observation period (28 days).Once successful weaning was achieved, the infant was recorded as an event. Recurrent respiratory deterioration requiring re-initiation of support after successful weaning was treated as a censoring event; however, such recurrence was rare in this cohort.

### Statistical analysis

All statistical analyses were performed using R version 4.3.1. Continuous variables were assessed for normality and expressed as mean ± standard deviation (SD) for normally distributed data, or as median (interquartile range, IQR) for non-normally distributed data. Categorical variables were summarized as frequencies and percentages.

#### Collinearity diagnosis and variable preprocessing

Prior to model construction, Pearson correlation analysis was conducted among all candidate predictors to assess multicollinearity. Variables with absolute correlation coefficients |r| > 0.75 were deemed severely collinear; in such pairs, the variable with greater clinical relevance or fewer missing values was retained, while the redundant variable was excluded to ensure model stability.

Least Absolute Shrinkage and Selection Operator (LASSO) Cox regression with 10-fold cross-validation was employed for variable selection. Respiratory support duration (days) served as the survival time variable, and successful weaning served as the event status variable (event = 1, censored = 0). The optimal penalty parameter *λ* was determined through 10-fold cross-validation of partial likelihood deviance. Rather than strictly adhering to *λ*.min (the value minimizing cross-validated error) or *λ*.1se (the most parsimonious model within one standard error of the minimum), we adopted an empirical compromise strategy: we identified a *λ* value between *λ*.1se and *λ*.min that retained approximately ten predictors. This threshold balances predictive accuracy with clinical interpretability and aligns with published recommendations that nomograms exceeding 10–12 variables become impractical for routine bedside use.

The LASSO-selected variables were entered into a multivariable Cox proportional hazards regression model to estimate hazard ratios (HRs) and 95% confidence intervals (CIs). The proportional hazards (PH) assumption was evaluated using Schoenfeld residual tests (both global and per-variable).

#### Model discrimination and internal validation

Model discrimination was quantified using Harrell's concordance index (C-index). Time-dependent receiver operating characteristic (ROC) curves were constructed to calculate the area under the curve (AUC) for predicting successful weaning at postnatal days 3, 5, and 8. To correct for overfitting bias, bootstrap internal validation with 1000 resamples was performed to calculate the optimism-corrected C-index.

#### Model calibration

Calibration was assessed using optimism-corrected calibration curves derived from 500 bootstrap resamples at postnatal days 3, 5, and 8. Predicted probabilities of successful weaning were plotted against observed probabilities, and mean absolute prediction error (MAPE) was calculated at each time point. Prediction accuracy was further quantified using the Brier score at postnatal days 3, 5, 8, 14, 21, and 28; the integrated Brier score (IBS) was computed over the entire 28-day follow-up period.

#### Clinical utility evaluation

Decision curve analysis (DCA) was performed at postnatal days 5 and 8 to evaluate the net benefit of using the nomogram to guide weaning decisions across a range of threshold probabilities, compared with “treat-all” and “treat-none” strategies.

#### SHAP interpretability analysis

To enhance mechanistic transparency, SHAP (SHapley Additive exPlanations) analysis was performed using the shapviz package (version 0.9.0). For the Cox proportional hazards model, SHAP values were calculated based on the linear predictor (log hazard ratio scale, *η* = X*β*) rather than time-specific survival probabilities. This approach was chosen because the Cox model's partial likelihood depends on the ranking of linear predictors, and SHAP decompositions on the log-hazard scale provide time-invariant, additive feature contributions that are independent of the unspecified baseline hazard function. SHAP values were computed using the KernelSHAP approximation with Monte Carlo sampling (100 iterations per observation), which approximates exact Shapley values by sampling random feature coalitions and fitting a local weighted linear model. Global feature importance was quantified by the mean absolute SHAP value (mean |SHAP|) across the dataset. Individual predictions were decomposed using waterfall plots, and marginal effect patterns were visualized using dependence plots.

#### Sensitivity analyses

To verify the stability of findings in light of PH assumption violations, a series of sensitivity analyses were conducted: (1) a stratified Cox model allowing separate baseline hazard functions by NCPAP stratum; (2) a time-dependent coefficient Cox model incorporating an NCPAP   ×   log(time) interaction term to characterize the temporal evolution of the NCPAP effect; (3) exclusion of LDH/HBDH outliers (>99th percentile, *n* = 357); (4) restriction to a ≤ 14-day follow-up window (*n* = 352); (5) five-fold cross-validation to assess model stability. All reported *P*-values were two-sided, and *P* < 0.05 was considered statistically significant.

## Results

### Baseline characteristics

A total of 365 late preterm infants were included: 191 males (52.3%) and 174 females (47.7%); 155 multiple pregnancies (42.5%) and 210 singleton pregnancies (57.5%); 93 (25.5%) received pulmonary surfactant (PS), and 174 (47.7%) received NCPAP. Median gestational age was 35.0 (34.0, 36.0) weeks, birth weight 2,370.0 (2,130.0, 2,660.0) g, and 5-minute Apgar score 10.0 (9.0, 10.0). Baseline demographic and clinical characteristics are summarized in Table 1, and the multivariable Cox regression results are presented in Table 2.

**Table 1:** Baseline characteristics of late preterm infants (*n* = 365).

Variable	Value	Distribution
AGE(*n* = 365)	35.0 (34.0, 36.0)	Non-normally distributed
Apgar_1(*n* = 365)	9.0 (8.0, 9.0)	Non-normally distributed
Apgar_10(*n* = 365)	10.0 (9.0, 10.0)	Non-normally distributed
Apgar_5(*n* = 365)	10.0 (9.0, 10.0)	Non-normally distributed
BW(*n* = 365)	2,370.0 (2,130.0, 2,660.0)	Non-normally distributed
M_GGT(*n* = 365)	10.0 (7.0, 19.0)	Non-normally distributed
M_ALP(*n* = 365)	127.0 (95.0, 171.0)	Non-normally distributed
M_LDH(*n* = 365)	197.0 (152.0, 254.0)	Non-normally distributed
PH(*n* = 365)	7.3 (7.3, 7.3)	Non-normally distributed
PCO2(*n* = 365)	45.7 (39.9, 53.0)	Non-normally distributed
PO2(*n* = 365)	82.0 (67.2, 105.0)	Non-normally distributed
HCO3(*n* = 365)	22.2 (20.1, 24.2)	Non-normally distributed
Lac(*n* = 365)	2.0 (1.4, 2.6)	Non-normally distributed
PCT(*n* = 365)	0.1 (0.1, 0.2)	Non-normally distributed
WBC(*n* = 365)	11.1 (8.9, 13.3)	Non-normally distributed
NEUT_PC(*n* = 365)	46.3 (38.2, 54.7)	Non-normally distributed
LYM_PC(*n* = 365)	42.9 (35.5, 51.2)	Non-normally distributed
RDW(*n* = 365)	15.4 (14.9, 16.1)	Non-normally distributed
PDW(*n* = 365)	16.4 (16.2, 16.6)	Non-normally distributed
HGB(*n* = 365)	173.7 ± 18.1	Normally distributed
HCT(*n* = 365)	0.5 ± 0.1	Normally distributed
PLT(*n* = 365)	261.5 ± 61.7	Normally distributed
Hs_CRP(*n* = 365)	0.1 (0.0, 0.2)	Non-normally distributed
INR(*n* = 365)	1.3 (1.2, 1.4)	Non-normally distributed
Fib(*n* = 365)	1.3 (1.1, 1.5)	Non-normally distributed
D_D(*n* = 365)	2.2 (1.2, 5.0)	Non-normally distributed
CK-MB(*n* = 365)	110.0 (82.0, 159.0)	Non-normally distributed
LDH(*n* = 365)	446.0 (387.0, 568.0)	Non-normally distributed
HBDH(*n* = 365)	261.0 (223.0, 317.0)	Non-normally distributed
A/G(*n* = 365)	2.3 (2.1, 2.7)	Non-normally distributed
GGT(*n* = 365)	161.0 (108.0, 239.0)	Non-normally distributed
ALP(*n* = 365)	152.0 (119.0, 186.0)	Non-normally distributed
SOD(*n* = 365)	154.0 (143.0, 167.0)	Non-normally distributed
PS(*n* = 365)	0 = 272 (74.5%); 1 = 93 (25.5%)	Categorical variable
NC(*n* = 365)	0 = 161 (44.1%); 1 = 204 (55.9%)	Categorical variable
SEX(*n* = 365)	0 = 174 (47.7%); 1 = 191 (52.3%)	Categorical variable
NCPAP(*n* = 365)	0 = 191 (52.3%); 1 = 174 (47.7%)	Categorical variable
MP(*n* = 365)	0 = 210 (57.5%); 1 = 155 (42.5%)	Categorical variable
SIMV(*n* = 365)	0 = 342 (93.7%); 1 = 23 (6.3%)	Categorical variable
WAY(*n* = 365)	0 = 54 (14.8%); 1 = 311 (85.2%)	Categorical variable
MSAF(*n* = 365)	0 = 351 (96.2%); 1 = 9 (2.5%); 2 = 3 (0.8%); 3 = 1 (0.3%); 4 = 1 (0.3%)	Categorical variable
PROM(*n* = 365)	0 = 264 (72.3%); 1 = 80 (21.9%); 2 = 21 (5.8%)	Categorical variable
PA(*n* = 365)	0 = 351(96.2%); 1 = 14(3.8%)	Categorical variable

**Table 2:** Multivariable Cox regression analysis of factors affecting respiratory support duration in late preterm infants (*n* = 365).HRs for LDH and HBDH are per 10-unit increase.

Variable	Coefficient	Wald Z	*P* value	HR (95% CI)
PS	−0.064	−0.45	0.652	0.938 (0.712–1.237)
AGE	0.09	1.24	0.217	1.094 (0.949–1.262)
Apgar_5	0.084	1.18	0.239	1.087 (0.946–1.250)
NCPAP	−0.352	−3.12	0.002	0.703 (0.564–0.878)
MP	0.254	2.30	0.022	1.289 (1.038–1.601)
LYM_PC	−0.013	−2.64	0.008	0.987 (0.978–0.997)
LDH	−0.001	−2.48	0.013	0.991 (0.984–0.998)
HBDH	−0.001	−1.86	0.062	0.988 (0.975–1.001)
A/G	0.122	1.97	0.049	1.130 (1.001–1.275)
SOD	0.014	5.21	<0.001	1.014 (1.009–1.019)

### Variable selection and collinearity diagnosis

**Figure 1:**
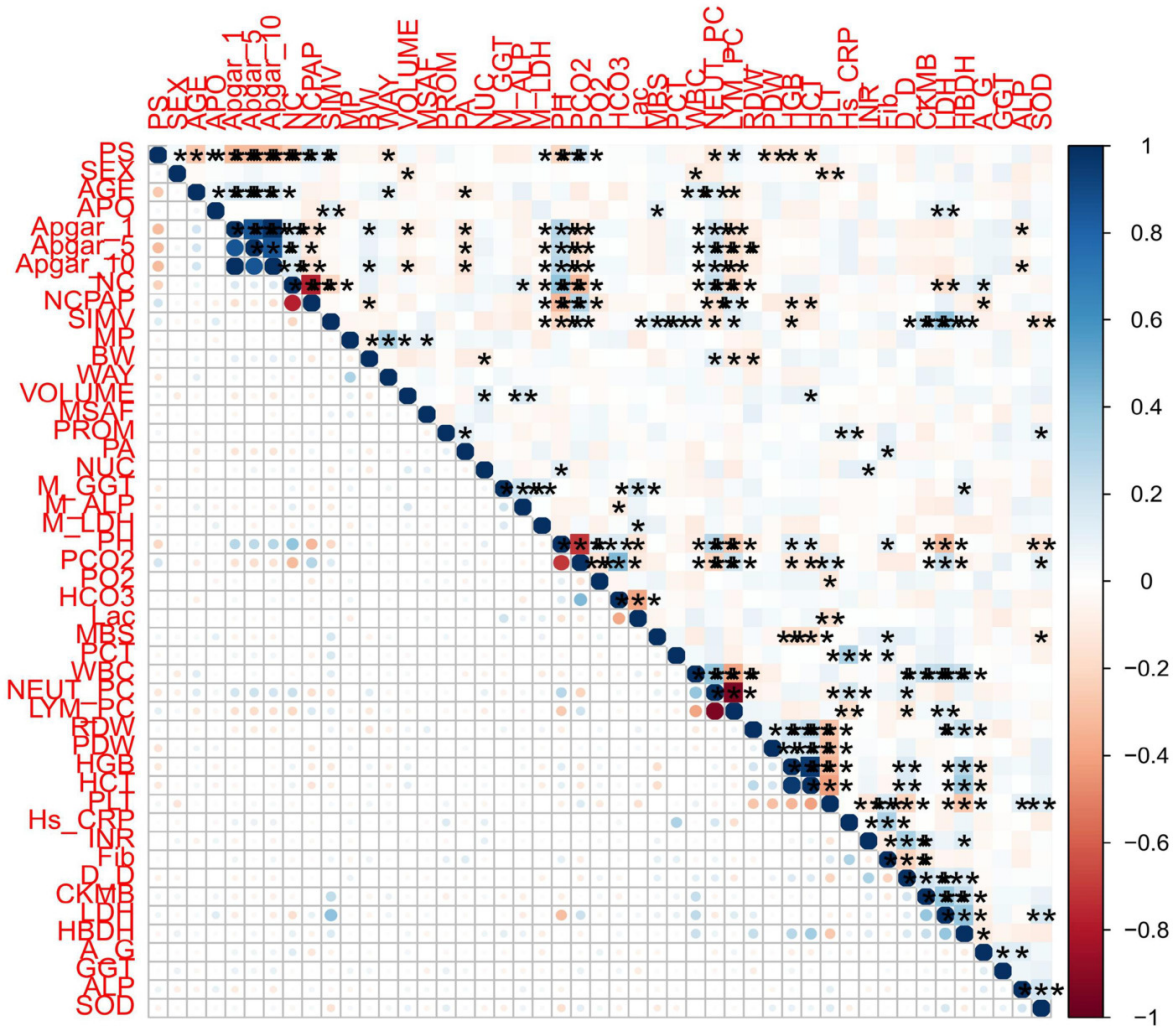
Variable collinearity heatmap and correlation coefficient matrix. Displays Pearson correlation coefficients among candidate variables. Red indicates positive correlation, blue indicates negative correlation.

**Figure 2:**
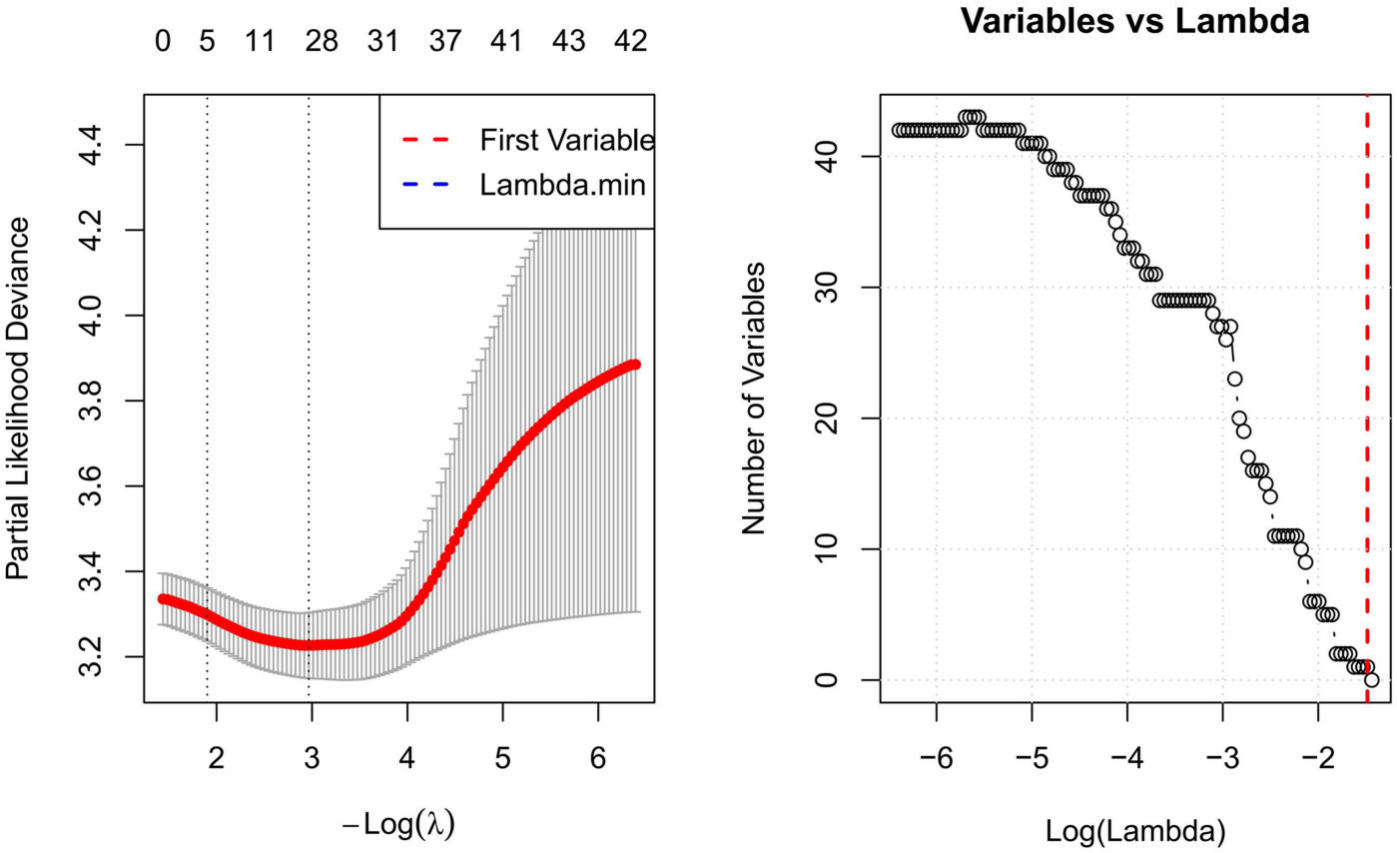
LASSO regression coefficient paths and 10-fold cross-validation curves. Coefficient paths showing predictor trajectories across log(*λ*). Cross-validation error curve; the vertical dashed line indicates the empirically selected *λ* = 0.118 (retaining 10 variables), balancing model parsimony with predictive accuracy.

Before building the predictive model, multicollinearity among variables was checked using Pearson correlation. The correlation matrix is visualized in Figure 1, and the LASSO coefficient paths and cross-validation curves are shown in Figure 2. Variables with |r| > 0.75 were deemed collinear, and the one with more clinical relevance or fewer missing values was kept. High multicollinearity was found between 1-minute and 10-minute Apgar scores (r = 1.00), 1-minute and 5-minute Apgar scores (r = 0.85), and between nasal cannula oxygen therapy and nasal continuous positive airway pressure (r = −0.78). To ensure model stability, the 1-minute and 10-minute Apgar scores and nasal cannula were removed. The remaining variables were then used in the LASSO-Cox regression analysis.

### LASSO regression variable selection

Least absolute shrinkage and selection operator (LASSO) regression with 10-fold cross-validation was employed for variable selection. Prior to LASSO, three variables exhibiting multicollinearity with other predictors (Apgar_1, Apgar_10, and Nasal Cannula Oxygen Therap) were removed to ensure independent contributions. The remaining 44 candidate variables were entered into the LASSO-Cox model. In standard practice, lambda.min (the value minimizing cross-validated partial likelihood deviance) retained 29 predictors, which we considered excessive for a bedside clinical nomogram and risked overfitting in this cohort. Conversely, lambda.1se (the most parsimonious model within one standard error of the minimum) retained only 7 variables, omitting several predictors with established clinical relevance to weaning physiology (e.g., Apgar_5, lymphocyte percentage, and acid-base status). To balance predictive accuracy with clinical interpretability, we adopted an empirical compromise strategy: we identified the lambda value within the interval between lambda.1se and lambda.min that retained approximately ten predictors. This threshold aligns with published recommendations that nomograms exceeding 10–12 variables become impractical for routine clinical use. The final multivariable Cox proportional hazards model was constructed using these LASSO-selected variables: PS, AGE, Apgar_5, NCPAP, MP, LYM_PC, LDH, HBDH, A/G, and SOD. Internal validation and stability assessment. Bootstrap resampling (*n* = 500) confirmed a stratified stability profile. Four core predictors demonstrated high stability (bootstrap frequency >80%): SOD (100.0%), NCPAP (91.6%), LDH (85.6%), and HBDH (83.4%). Six auxiliary predictors showed moderate stability (50%–80%): LYM_PC (78.2%), A/G (68.2%), MP (65.2%), RDW (60.8%), Apgar_5 (53.4%), and AGE (52.8%). The mean bootstrap selection frequency across all ten variables was 72.9%. Optimism-corrected performance metrics were derived from these resamples (Supplementary Table S1).

### Multivariable Cox model

Ten LASSO-selected variables were entered into the multivariable Cox proportional hazards model. The model demonstrated good overall fit: likelihood ratio test *χ*^2^ = 84.68, *P* < 0.001; Wald test *χ*^2^ = 79.47, *P* < 0.001. Schoenfeld residual analysis showed that the global test was significant $\chi^{2}=26.70,P=0.003$, indicating violation of the proportional hazards assumption for the model as a whole. Time-varying effects were observed for NCPAP $\chi^{2}=9.77,P=0.002$, LDH $\chi^{2}=5.13,P=0.023$, and HBDH $\chi^{2}=5.13,P=0.023$. To verify the stability of our findings in light of these violations, we conducted a series of sensitivity analyses (Table 3, Supplementary Table S2).

**Table 3:** Sensitivity analyses for the Cox proportional hazards model.HR for LDH is per 10-unit increase.

Variable	Primary Cox HR (95% CI)	Stratified Cox HR (95% CI)	Time-Dependent Cox HR (95% CI)	Excluding Outliers HR (95% CI)	≤14 days HR (95% CI)
NCPAP	0.703 (0.564–0.878)	Stratified	0.576 (0.398–0.834)†	0.708 (0.560–0.896)	0.752 (0.585–0.967)
MP	1.289 (1.038–1.601)	1.295 (1.043–1.607)	1.206 (0.972–1.497)	1.270 (1.019–1.583)	1.291 (1.024–1.628)
SOD	1.014 (1.009–1.019)	1.014 (1.008–1.019)	1.012 (1.007–1.018)	1.014 (1.009–1.020)	1.011 (1.006–1.017)
LYM\_PC	0.987 (0.978–0.997)	0.987 (0.978–0.997)	0.987 (0.978–0.997)	0.986 (0.977–0.996)	0.989 (0.979–0.999)
LDH	0.991 (0.984–0.998)	0.992 (0.985–0.999)	0.992 (0.985–0.999)	0.992 (0.984–1.000)	0.991 (0.983–0.998)
A/G	1.130 (1.001–1.275)	1.103 (0.975–1.247)	1.181 (1.047–1.333)	1.121 (0.991–1.268)	1.097 (0.965–1.247)

First, a stratified Cox model allowing separate baseline hazard functions by NCPAP stratum was fitted to eliminate the PH violation for the strongest offending variable. The hazard ratios for the remaining predictors were virtually identical to the primary model (maximum difference < 0.006), including MP (HR = 1.295, 95% CI: 1.043–1.607, *P* = 0.019), SOD (HR = 1.014, 95% CI: 1.008–1.019, *P* < 0.001), LYM_PC (HR = 0.987, 95% CI: 0.978–0.997, *P* = 0.011), and LDH (HR = 0.991, 95% CI: 0.984–0.998, *P* = 0.018; Table 3). Second, a time-dependent coefficient Cox model incorporating an NCPAP   ×   log(time) interaction term confirmed a significant temporal evolution of the NCPAP effect (interaction HR = 0.085, 95% CI: 0.060–0.121, *P* < 0.001), with the NCPAP hazard ratio transitioning from 79.2 at postnatal day 1 to 0.58 at day 8 (Supplementary Table S3). This pattern reflects early severity indication confounding (physicians selecting NCPAP for initially sicker infants) followed by mid-term protective effect once acute perinatal instability resolves, which is consistent with the ascending time-dependent AUC observed in our discrimination analysis. Third, additional sensitivity analyses excluding LDH/HBDH outliers (>99th percentile, *n* = 357) or restricting to a ≤ 14-day follow-up window n=352 confirmed the stability of the SOD and NCPAP effects (Table 3, Supplementary Table S2). Five-fold cross-validation yielded a mean C-index of 0.669 (±0.022), nearly identical to the original C-index of 0.678, further supporting model stability. Taken together, these analyses demonstrate that the principal prognostic conclusions are robust to PH assumption violations.

**Figure 3:**
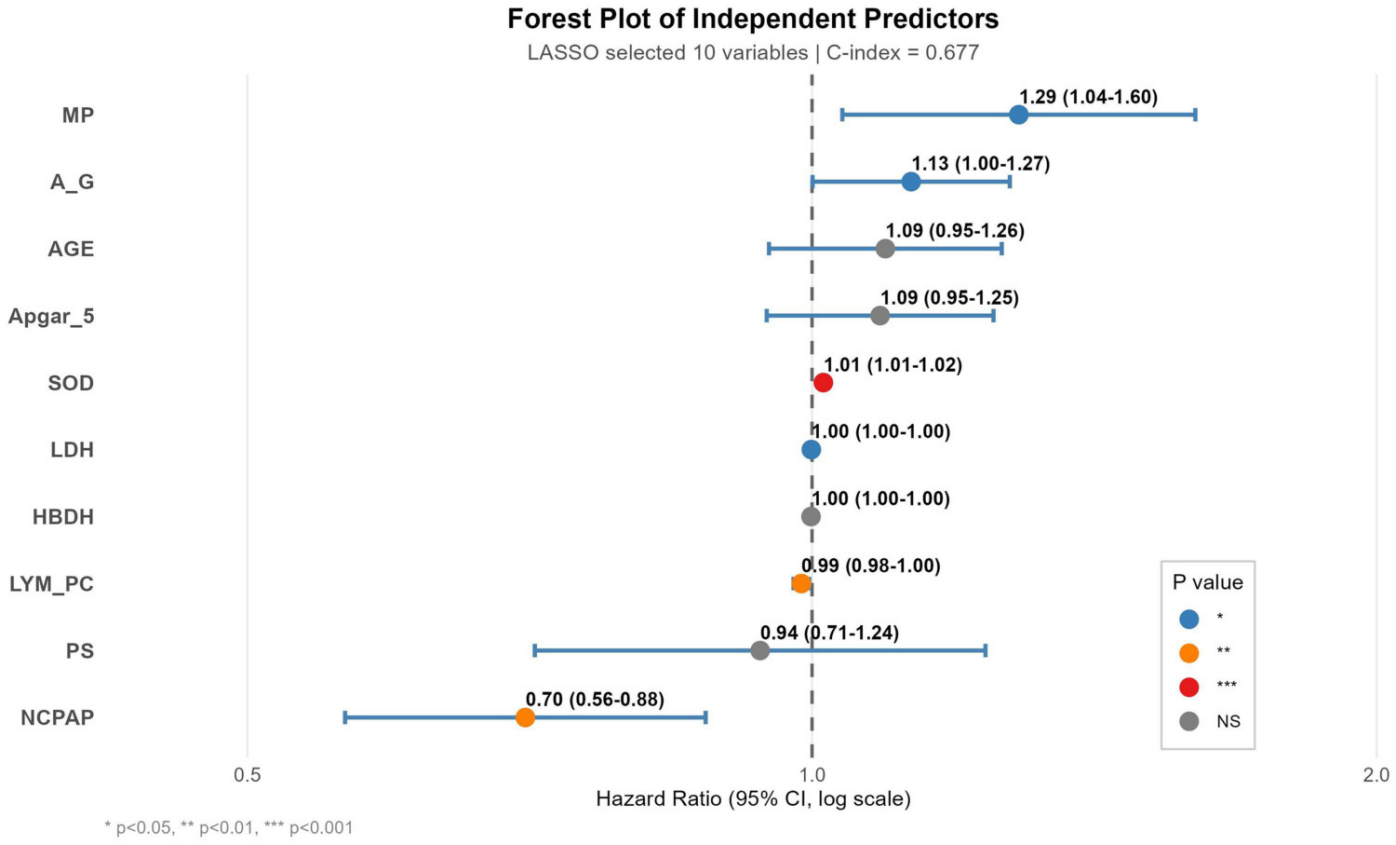
Multivariable Cox regression forest plot. Illustrating the impact of ten retained variables on respiratory support duration in late preterm infants. Squares represent hazard ratios (HRs), and horizontal lines indicate 95% confidence intervals. HR < 1 (to the left of the vertical dashed reference line) denotes protective factors facilitating successful weaning; HR > 1 (to the right of the vertical dashed reference line) denotes risk factors prolonging oxygen dependency. A *P*-value <0.05 was considered statistically significant.

The multivariable Cox regression forest plot (Figure 3) illustrates the effects of the ten retained variables on respiratory support duration in late preterm infants, visually presenting the magnitude of effect sizes and their confidence intervals. NCPAP and SOD demonstrated the most significant effects (*P* < 0.01), while MP, LYM_PC, LDH, and A/G also showed independent predictive value. In contrast, PS, AGE, and Apgar_5 displayed 95% confidence intervals crossing 1.0 (non-significant).

**Figure 4:**
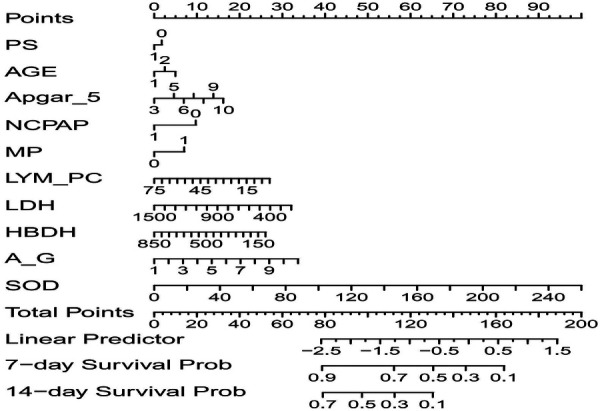
Nomogram for predicting successful weaning in late preterm infants. Constructed from Cox regression coefficients for individualized probability estimation at postnatal days 7 and 14. For each variable, draw a vertical line upward to the “Points” axis to obtain the score; sum all points and locate the total on the “Total Points” axis; project downward to read the predicted probability.

A nomogram predictive model was constructed based on Cox regression coefficients to enable individualized assessment of weaning risk (Figure 4). The nomogram incorporated ten predictors (PS, AGE, Apgar_5, NCPAP, MP, LYM_PC, LDH, HBDH, A/G, and SOD), with each variable assigned corresponding points derived from its regression coefficient. The summed total points correspond to predicted probabilities of successful weaning at postnatal days 7 and 14.

### Model performance

**Figure 5:**
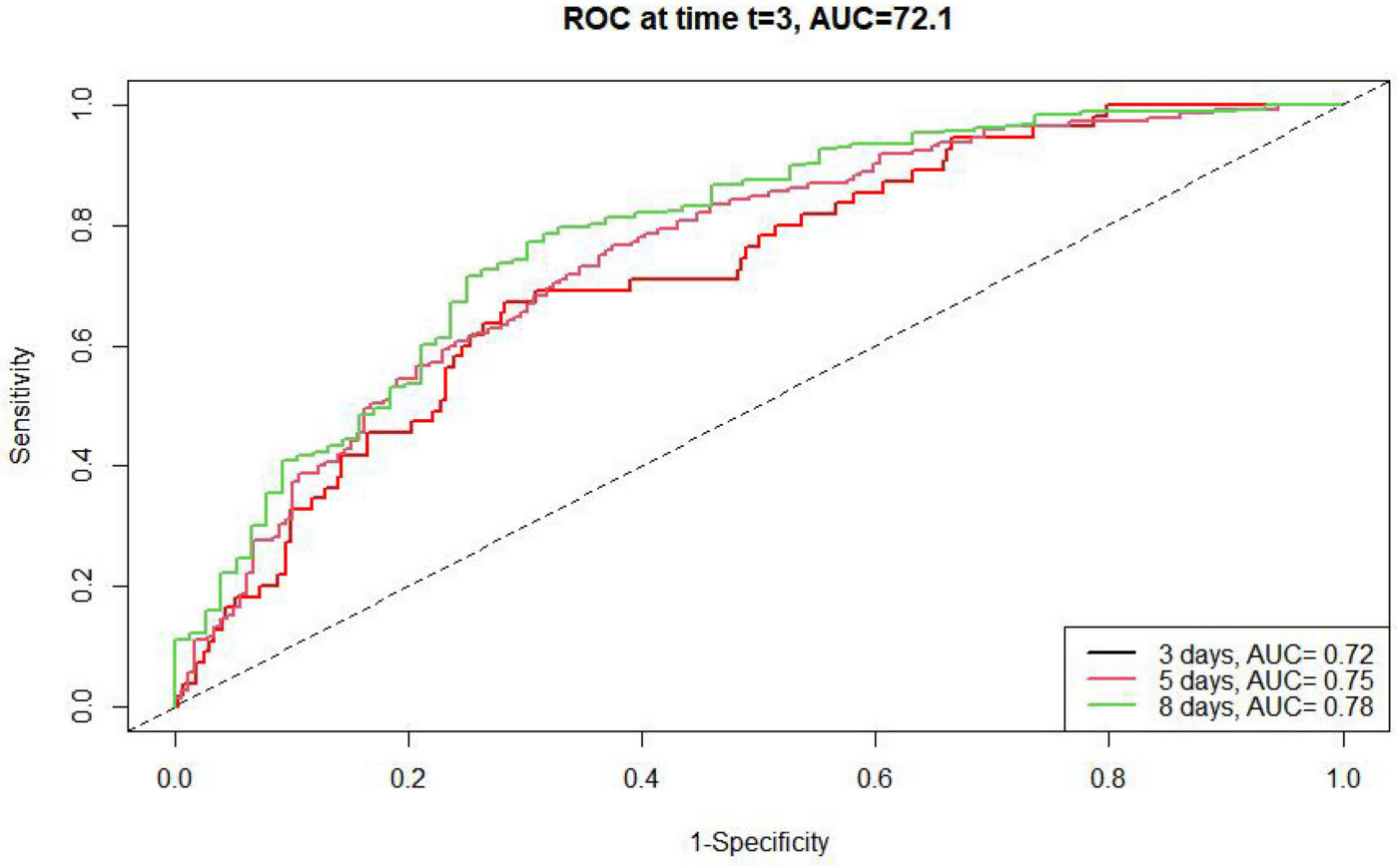
Time-dependent receiver operating characteristic (ROC) curves. Evaluated at three time points (postnatal days 3, 5, and 8) to assess the model's discriminative ability. Area under the curve (AUC) values increased from 0.732 (95% CI: 0.662–0.801) at day 3 to 0.782 (95% CI: 0.720–0.844) at day 8, indicating good predictive performance of the model for mid-term weaning outcomes.

Time-dependent receiver operating characteristic (ROC) curves were used to evaluate the model's discriminative ability for weaning outcomes at various time points. As shown in Figure 5, predictive accuracy improved progressively over time: the area under the curve (AUC) for predicting successful weaning at postnatal day 3 was 0.732 (95% CI: 0.662–0.801), at day 5 was 0.769 (95% CI: 0.718–0.820), and at day 8 reached 0.782 (95% CI: 0.720–0.844). According to standard discrimination criteria, the model demonstrated acceptable predictive performance at day 3 (AUC > 0.70) and good performance at days 5 and 8 (AUC > 0.75), indicating stable predictive value for mid-term weaning outcomes in late preterm infants.

**Figure 6:**
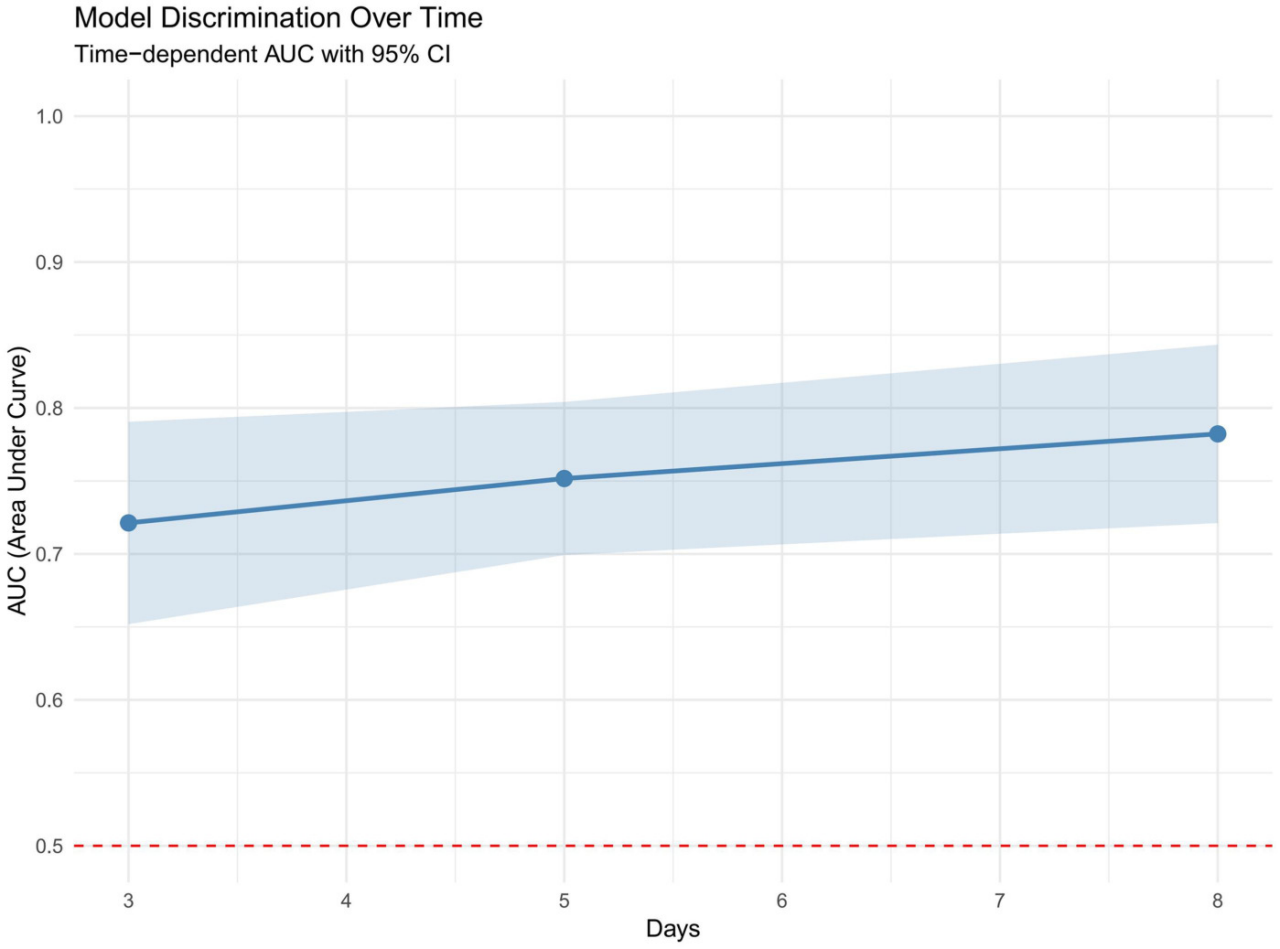
Dynamic trends in time-dependent discriminative ability. Temporal evolution of model discrimination across postnatal days 3, 5, and 8. The blue solid line connects td-AUC values (0.732, 0.769, and 0.782, respectively), with light blue shading indicating 95% confidence intervals. The red dashed line denotes the random chance level (AUC = 0.5).

The dynamic trend of time-dependent AUC (Figure 6) revealed that model discrimination improved progressively with extended follow-up (from day 3 to day 8, AUC increased from 0.732 to 0.782). AUC values at all specific time points exceeded the overall C-index (0.677), suggesting that the model's time-point-specific prediction outperformed its global risk ranking capability.

**Figure 7:**
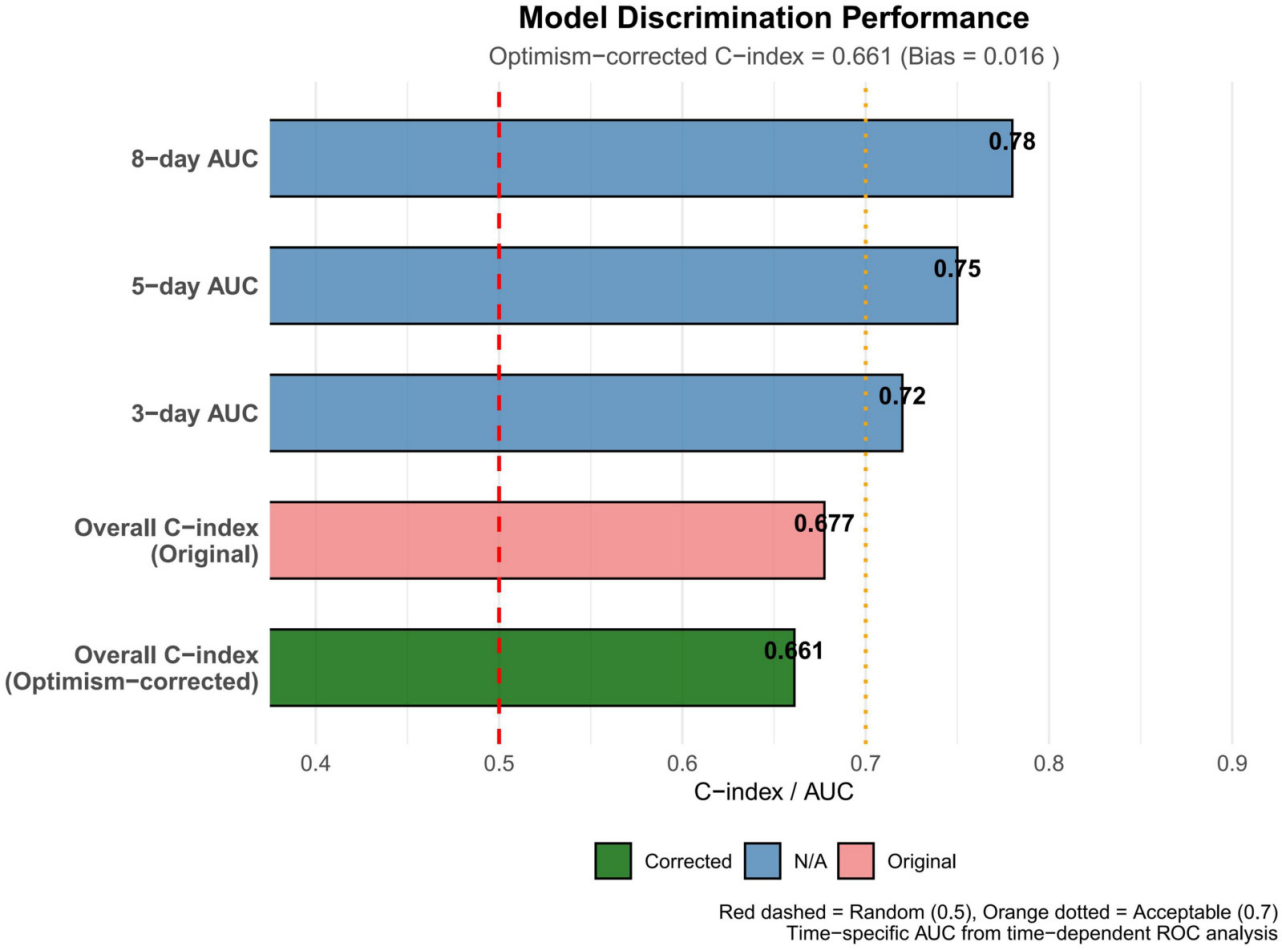
Summary of model discrimination evaluation metrics. Comprehensive presentation of time-dependent area under the curve (td-AUC) at three time points, the original C-index, and the optimism-corrected C-index following 1,000 bootstrap resamplings. AUC values of 0.7–0.8 indicate acceptable to good discriminative ability, while 0.6–0.7 indicate moderate discrimination.

Model discrimination was further assessed using the C-index with bootstrap internal validation. As illustrated in Figure 7, the original C-index was 0.677, which corrected to 0.661 after 1,000 bootstrap resamplings (optimism = 0.016), indicating moderate discrimination with good stability upon internal validation. Time-dependent AUC analysis confirmed an ascending discriminative trend across evaluation time points, with all specific time-point values exceeding the overall C-index, further confirming that focused time-point prediction is more accurate than overall risk stratification.

**Figure 8:**
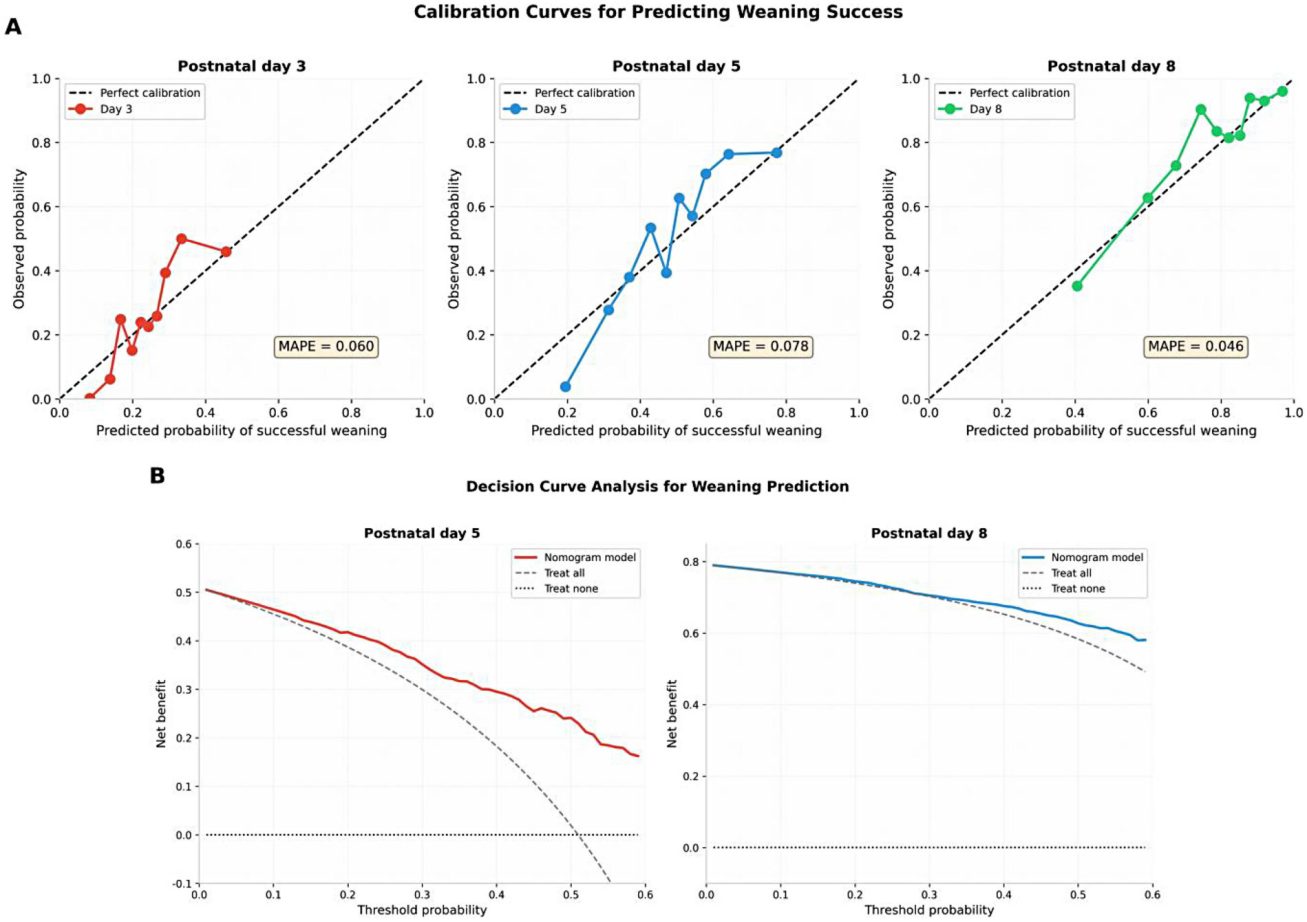
Optimism-corrected calibration curves and decision curve analysis for the nomogram model. **(A)** Calibration curves at postnatal days 3, 5, and 8 following 500 bootstrap resamplings. The dashed diagonal line denotes perfect calibration. **(B)** Decision curve analysis at days 5 and 8 comparing net benefit of the nomogram (solid lines) against treat-all (dashed) and treat-none (dotted) strategies across threshold probabilities.

Beyond discrimination, we assessed model calibration using optimism-corrected calibration curves with 500 bootstrap resamples at postnatal days 3, 5, and 8 (Figure 8A). The predicted probabilities showed close agreement with observed outcomes across all evaluated time points, with mean absolute calibration errors of 6.0%, 7.8%, and 4.6% at days 3, 5, and 8, respectively. Prediction accuracy was further quantified using the Brier score, which yielded 0.184, 0.176, 0.169, 0.163, 0.159, and 0.156 at postnatal days 3, 5, 8, 14, 21, and 28, respectively, with an integrated Brier score (IBS) of 0.171 over the 28-day follow-up period.

Clinical utility was evaluated using decision curve analysis (DCA) at postnatal days 5 and 8 (Figure 8B). At day 5, the nomogram model provided a higher net benefit than both the “treat-all” and “treat-none” strategies across clinically relevant threshold probability ranges of 0.05–0.59. At day 8, the model showed comparable net benefit to the “treat-all” strategy, reflecting the high weaning success rate (79.2%) observed at this time point; this limits incremental clinical utility when the majority of infants are already ready for extubation. Collectively, these metrics demonstrate that the nomogram achieves acceptable discrimination, satisfactory calibration, and incremental clinical value for guiding weaning decisions in late-preterm infants, with the greatest utility observed within the first postnatal week.

### SHAP interpretability analysis

**Figure 9:**
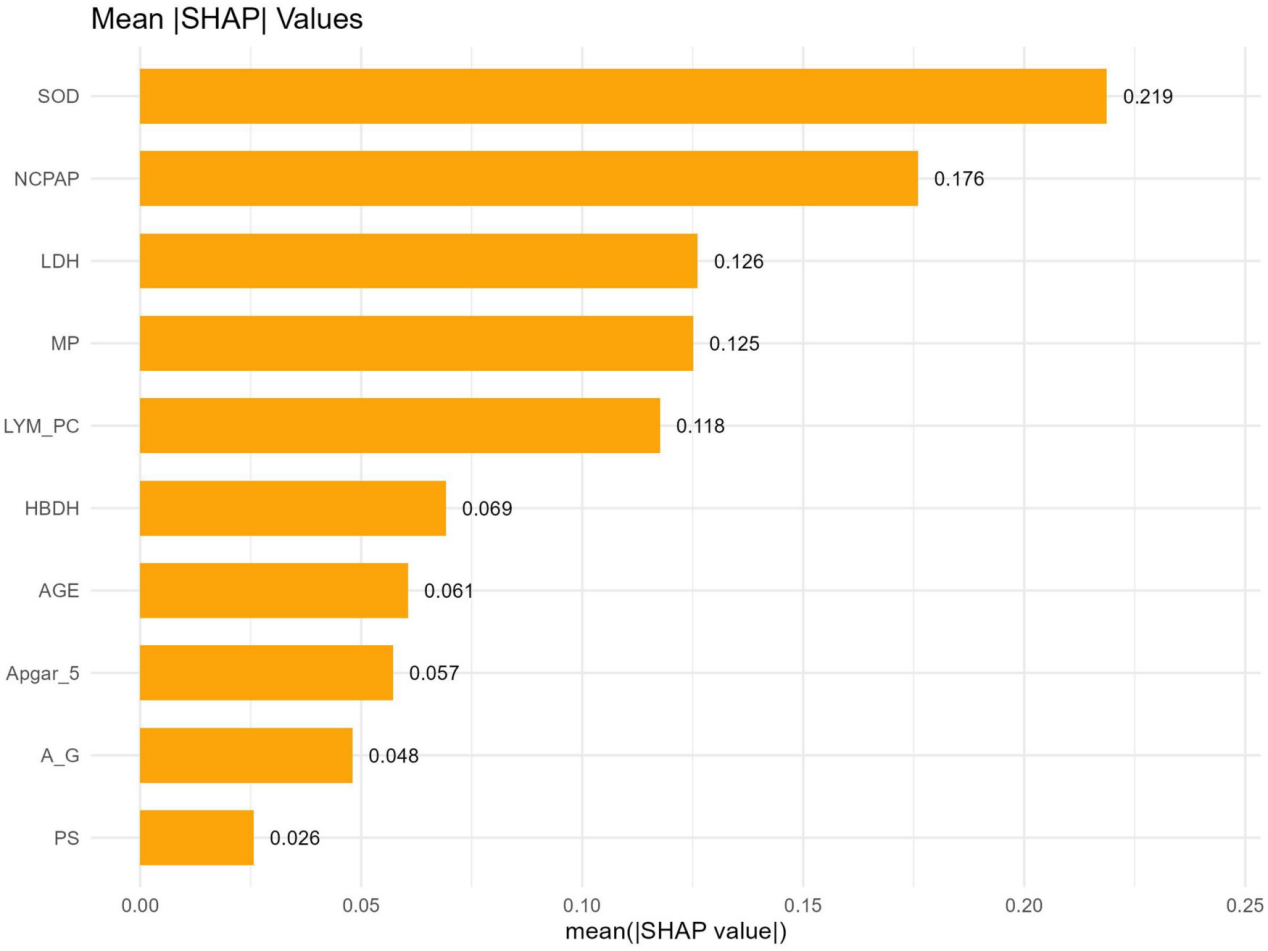
SHAP feature importance summary bar plot. Displays the mean absolute SHAP values (mean |SHAP|) for each variable, arranged in descending order of global importance. SOD (superoxide dismutase), NCPAP (nasal continuous positive airway pressure), and LYM_PC (lymphocyte percentage) were identified as the top three factors influencing model predictions, consistent with the Cox regression analysis results.

To elucidate the predictive mechanism of the model and verify clinical interpretability, SHAP (SHapley Additive exPlanations) analysis was employed to quantify the contribution of individual variables to personalized predictions. Global feature importance ranking demonstrated that SOD was the primary driver of model predictions (highest mean |SHAP| value), followed by NCPAP and LYM_PC, consistent with the magnitude and direction of effects observed in the Cox regression analysis, suggesting biologically plausible decision logic (Figure 9).

**Figure 10:**
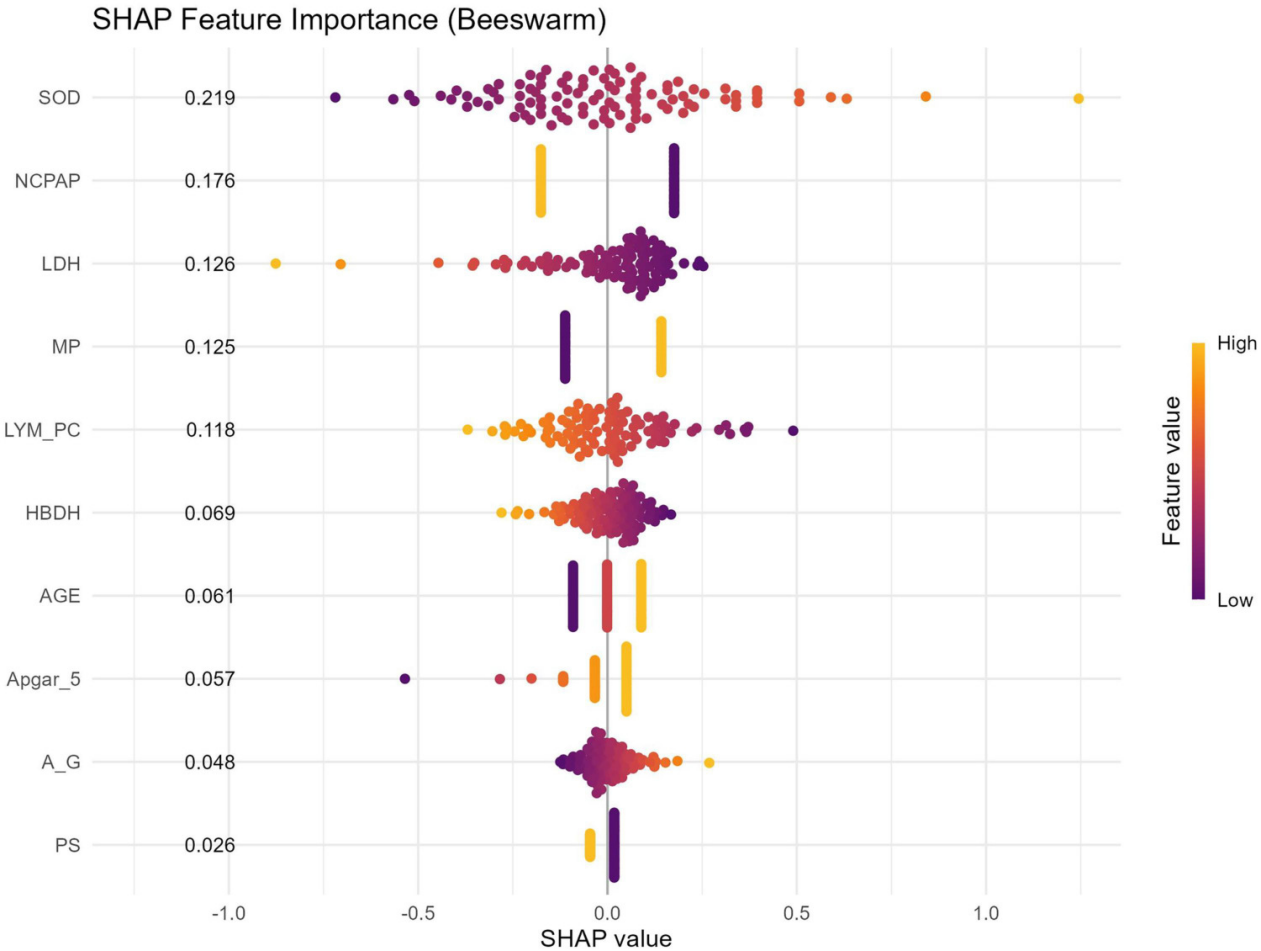
SHAP beeswarm plot. Illustrates the distribution of SHAP values for each variable across the dataset, where each point represents an individual sample. The color gradient (red = high feature values, blue = low feature values) indicates the direction of feature values. High SOD values (red dots) clustered predominantly in the positive SHAP region (indicating prolonged oxygen dependency), whereas NCPAP use (categorical variable, red = used) concentrated in the negative region (indicating shortened oxygen dependency).

The SHAP beeswarm plot (Figure 10) revealed dose-response relationships between feature values and risk contribution: SOD levels showed a marked positive distribution [high values (red) concentrated in positive SHAP regions], indicating that elevated oxidative stress significantly increased the risk of weaning delay. Conversely, NCPAP use (red points indicating usage) clustered predominantly in negative SHAP regions, demonstrating substantial protective effects of early non-invasive ventilation. High lymphocyte percentages (LYM_PC) (red) tended toward negative SHAP values, suggesting faster weaning in infants with robust immune function.

**Figure 11:**
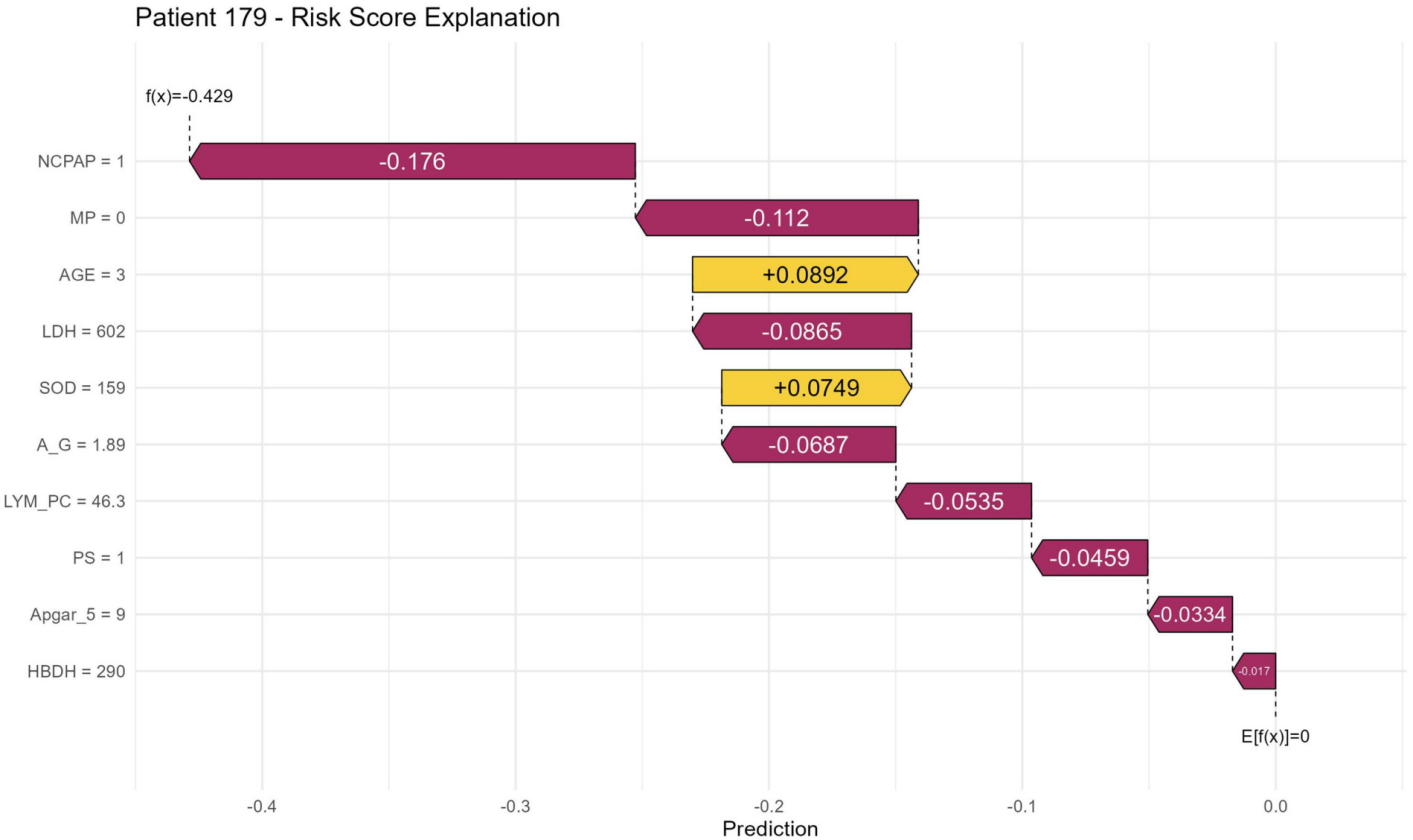
SHAP waterfall plot (representative case). Demonstrates the prediction decomposition for an individual representative case. The base value indicates the average model prediction across the dataset. Each variable pushes the prediction upward (red, indicating increased weaning failure risk or prolonged oxygen dependency) or downward (blue, indicating decreased risk or shortened oxygen dependency) according to its actual observed value, culminating in the final risk score (final value) for that individual.

Individual waterfall plots (Figure 11) illustrated the decomposition process of predictions for representative cases: starting from the base risk value, each variable pushed the prediction upward (red, indicating increased weaning failure risk or prolonged oxygen dependency) or downward (blue, indicating decreased risk or shortened duration) based on its observed value, cumulating to yield the personalized risk score (final value). For instance, an infant with high SOD levels, multiple pregnancy, and no NCPAP use showed prominent positive contributions from SOD and MP variables (red bars), while the absence of NCPAP failed to provide negative buffering, resulting in a final risk score substantially higher than average, indicating the need for intensified respiratory support management.

**Figure 12:**
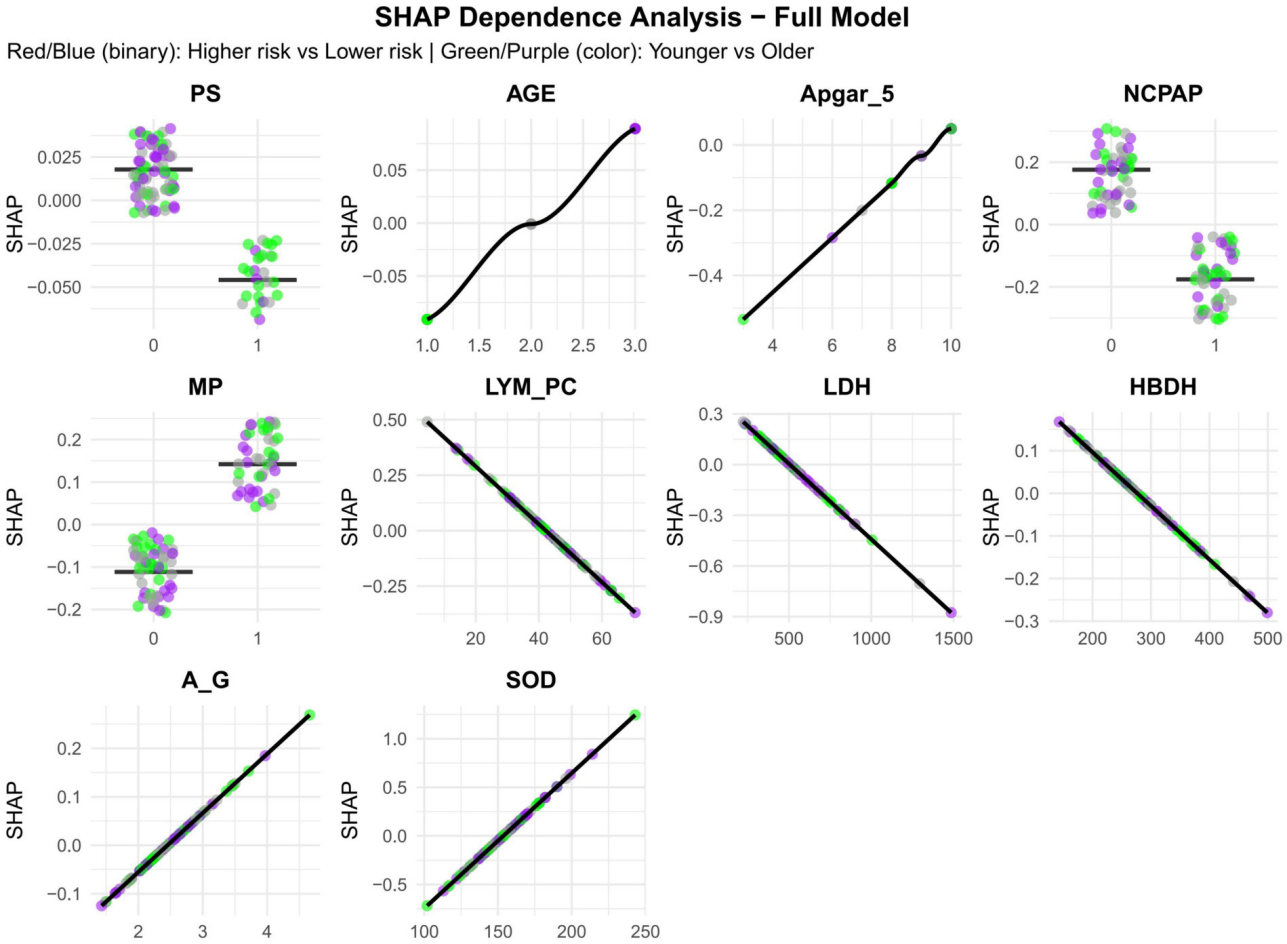
SHAP dependence plot. Each panel displays the relationship between the observed value of a predictor (*x*-axis) and its corresponding SHAP value (log-hazard contribution, *y*-axis). Color coding for interaction effects: The color gradient indicates the value of the interacting variable gestational age (AGE). Green represents younger infants; purple represents older infants; gray represents intermediate ages. The divergent color pattern demonstrates how the effect of the focal predictor on weaning prediction is modified across the spectrum of gestational age. Units: SOD [U/mL], LDH [U/L], HBDH [U/L], LYM_PC [%], A/G [ratio], Apgar_5 [score], RDW [%], AGE [weeks].

SHAP dependence plots (Figure 12) further illustrated marginal effect curves for key continuous variables (e.g., SOD), confirming that the positive contribution to weaning risk increased non-linearly with rising SOD levels, with risk increments becoming more pronounced when SOD exceeded 160 U/mL, providing a reference for establishing clinical intervention thresholds.

## Discussion

In this study, we enrolled 365 late preterm infants and, for the first time, integrated LASSO-Cox regression with SHAP interpretability analysis to systematically investigate the determinants of respiratory support duration. Unlike previous research that relied on single indicators or conventional logistic regression (6), our methodology employed the LASSO algorithm to effectively address multicollinearity in high-dimensional clinical data, thereby ensuring model parsimony while maintaining clinical relevance. Our results suggest that multiple pregnancies and elevated SOD levels are associated with prolonged respiratory support duration, whereas early application of NCPAP, higher lymphocyte percentages, and relatively lower LDH levels are conducive to earlier weaning. The developed nomogram exhibited moderate discrimination, with a C-index of 0.661 following bootstrap correction, and a time-dependent AUC ranging from 0.72 to 0.78, comparable to recent pediatric survival prediction models (7, 8).

### Multiple pregnancy and oxidative stress

Factors Contributing to Prolonged Respiratory Support. In our model, multiple pregnancy (MP) emerged as the most significant clinical risk factor (HR = 1.289), likely due to complications specific to multifetal gestations, such as twin-to-twin transfusion syndrome (TTTS) and selective intrauterine growth restriction (sIUGR), which adversely affect fetal lung development (9). Furthermore, multifetal pregnancies are often associated with placental insufficiency, chronic intrauterine hypoxia, and systemic inflammation, all of which collectively elevate the risk of respiratory distress syndrome (RDS) postnatally (10). Given that multiple pregnancies comprised 42.5% of our cohort, enhanced respiratory monitoring for this subgroup may be warranted, though prospective trials are needed to confirm whether targeted interventions modify outcomes.

The observation concerning superoxide dismutase (SOD) presents a mechanistic paradox. Traditionally, elevated SOD levels are indicative of a protective antioxidant response. However, our findings reveal a positive association between SOD levels and prolonged oxygen dependency (hazard ratio = 1.014, *P* < 0.001), with SOD emerging as the primary driver of model predictions according to SHAP analysis. We propose that this apparent “paradox” may be attributed to compensatory upregulation in response to severe oxidative stress: in situations where late preterm infants experience significant oxidative damage—due to hyperoxia exposure and inflammatory responses—SOD expression is upregulated to mitigate excess reactive oxygen species (ROS). Nevertheless, by this stage, pulmonary tissue may have already incurred irreversible damage, as evidenced by increased alveolar-capillary membrane permeability, pulmonary edema, and surfactant inactivation (11). This hypothesis is consistent with findings from perinatal hypoxic-ischemic injury models, where SOD upregulation is indicative of mitochondrial dysfunction rather than a protective capacity (12). Thus, elevated SOD likely signifies “stressed” rather than “protected” biological states, suggesting that clinicians should focus on minimizing oxidative exposure (optimal oxygen targeting, avoidance of hyperventilation) rather than relying solely on endogenous antioxidant responses.

### Respiratory support strategies: NCPAP and pulmonary surfactant

Notably, the utilization of nasal continuous positive airway pressure (NCPAP) significantly reduced the duration of respiratory support [hazard ratio (HR) = 0.703], whereas the administration of pulmonary surfactant (PS) did not demonstrate an independent correlation with the time required for weaning (*P* = 0.652). This finding is consistent with international neonatal resuscitation guidelines, which recommend prioritizing “non-invasive ventilation first” approaches (13). Late preterm infants, defined as those born between 34 and 36⁺⁶ weeks of gestation, exhibit relatively mature pulmonary structures and experience only mild surfactant deficiency. The early application of NCPAP helps to maintain functional residual capacity and prevents alveolar collapse, thereby avoiding the risks of ventilator-induced lung injury and secondary infections associated with intubation (14).

The null association between PS and weaning time should be interpreted cautiously. In this retrospective cohort, PS administration was likely confounded by unmeasured severity indicators—such as antenatal corticosteroid exposure, maternal chorioamnionitis, and initial ventilator settings—that were not fully captured. Infants receiving PS presumably had more severe RDS at baseline, and without adjustment for these confounders, the true biologic effect of PS may be masked by indication bias. Additionally, PS administration requires transient intubation, which may disrupt non-invasive ventilation continuity. These observational data suggest that prioritizing early NCPAP in late preterm infants may be a reasonable approach, though this hypothesis requires confirmation in randomized or prospective studies.

Notably, Schoenfeld residual analysis revealed a proportional hazards (PH) violation for NCPAP, which we addressed through stratified and time-dependent coefficient Cox models. The time-dependent analysis demonstrated that the NCPAP hazard ratio evolved from 79.2 on postnatal day 1 to 0.58 by day 8, reflecting probable early severity-indication confounding (clinicians selectively applying NCPAP to initially sicker infants) followed by mid-term protective effects once acute perinatal instability resolved. This temporal pattern corroborates the ascending td-AUC observed in our discrimination analysis and underscores the importance of time-stratified prediction in neonatal weaning research.

### Laboratory biomarkers

The laboratory indicators provided essential mechanistic insights. Specifically, lymphocyte percentage (LYM_PC), lactate dehydrogenase (LDH), and the albumin-to-globulin ratio (A/G) served as indicators of immune function, tissue injury, and nutritional-inflammatory status, respectively. An elevated LYM_PC (HR = 0.987, *P* = 0.008) suggests robust immune maturity, as lymphocytes contribute to alveolar epithelial repair through the secretion of anti-inflammatory cytokines (IL-10, TGF-*β*) and modulation of macrophage polarization (15). Reduced LDH levels (HR = 0.991, *P* = 0.013) are indicative of preserved alveolar-capillary membrane integrity and diminished parenchymal cellular injury, which may facilitate shorter durations of mechanical ventilation (16). In contrast, an increased A/G ratio (HR = 1.130, *P* = 0.049) could signify immunocompromise associated with hypogammaglobulinemia or hyperalbuminemia reflecting dehydration or inflammatory states, thereby impairing the capacity for pulmonary tissue repair (17). The integrative use of these three indices underscores the utility of LASSO-Cox modeling in providing a comprehensive prognostic assessment across immune, injury, and nutritional systems.

### Methodological innovation and model interpretability

This study offers two methodological contributions. Firstly, we utilized an empirical lambda selection strategy for LASSO-Cox regression, which balances the risk of omitting weak yet significant predictors (associated with lambda.1se) against the risk of overfitting (associated with lambda.min) (18) In line with recent applications in the prognostication of neonatal necrotizing enterocolitis (19), this approach adeptly handled high-dimensional clinical data while maintaining model parsimony, thereby addressing the limitations inherent in traditional stepwise regression when applied to small pediatric samples.

Secondly, we employed SHAP (SHapley Additive exPlanations) analysis to enhance the clinical interpretability of our model, addressing the “black box” limitations inherent in conventional predictive models (5). The SHAP framework facilitates the visualization of how specific variables influence individual risk predictions at both the population and individual levels. SHAP beeswarm plots illustrated dose-response relationships: elevated SOD levels were associated with positive SHAP values (indicating increased risk), whereas the use of NCPAP was linked to negative SHAP values (indicating protective effects). These insights provide intuitive mechanistic understanding that traditional Cox proportional hazards models are unable to offer.

In terms of predictive performance, time-dependent ROC analysis revealed an enhancement in accuracy with prolonged observation periods (AUC increased from 0.732 on day 3 to 0.782 on day 8), indicating superior efficacy in predicting mid-term as opposed to early weaning outcomes. This improvement likely reflects the stabilization of acute perinatal factors, such as delivery room resuscitation and early pulmonary adaptation, by the intermediate phase, thereby facilitating a more reliable evaluation of the intrinsic capacity for respiratory recovery. Although the C-index of 0.661 suggests only moderate discrimination, this level of performance is deemed acceptable given the multifactorial nature of late preterm respiratory support, which involves complex interactions among genetic, environmental, and therapeutic factors (6). In alignment with established validation protocols for pediatric prediction models, bootstrap internal validation using 1,000 resamples demonstrated minimal optimism (0.016), thereby confirming robust internal validity.

### Limitations and future directions

This study possesses several limitations. Firstly, the retrospective design conducted at a single center may introduce selection bias, necessitating multicenter external validation to enhance generalizability. Secondly, although the event-per-variable ratio (EPV ≈ 36.5:1) surpassed the ideal thresholds (≥20:1), bootstrap internal validation cannot fully substitute for external validation, potentially limiting prediction accuracy for infrequent subgroups, such as those with extremely low birth weight. Thirdly, the lack of comprehensive documentation of specific therapeutic parameters, including the timing of pulmonary surfactant administration, initial settings of nasal continuous positive airway pressure (NCPAP), and weaning protocols, may introduce implementation bias. Lastly, the study did not account for potential confounding variables, such as the dosage of antenatal corticosteroids and the maternal prenatal infection status.

Future research endeavors should focus on conducting prospective multicenter cohort validations in accordance with established reporting standards for machine learning-based pediatric prognostic models, ensuring compliance with the TRIPOD-AI guidelines. Furthermore, incorporating dynamic longitudinal biomarkers, especially oxidative stress indicators measured serially throughout hospitalization, could significantly improve predictive accuracy beyond initial baseline assessments. Investigating targeted antioxidant interventions predicated on specific SOD thresholds (e.g., SOD >160 U/mL) offers a promising direction for interventional studies.

## Conclusion

The prediction model based on LASSO-Cox regression demonstrated moderate to good discrimination for respiratory support duration in late preterm infants. Multiple pregnancy and high oxidative stress levels (elevated SOD) are important warning signs for prolonged oxygen requirement, while early NCPAP intervention can effectively shorten the respiratory support period. The nomogram model combined with SHAP interpretability analysis provides a quantitative basis for individualized weaning assessment at the bedside in NICU.

## Data Availability

The original contributions presented in the study are included in the article/Supplementary Material, further inquiries can be directed to the corresponding author.
