# Supplementary figures and images for: Risk factors for prolonged respiratory support in late preterm infants: a LASSO-Cox regression analysis
Source: Front Pediatr. 2026 Jun 10;14:1832233. doi: 10.3389/fped.2026.1832233 (PMC13290591; doi:10.3389/fped.2026.1832233)

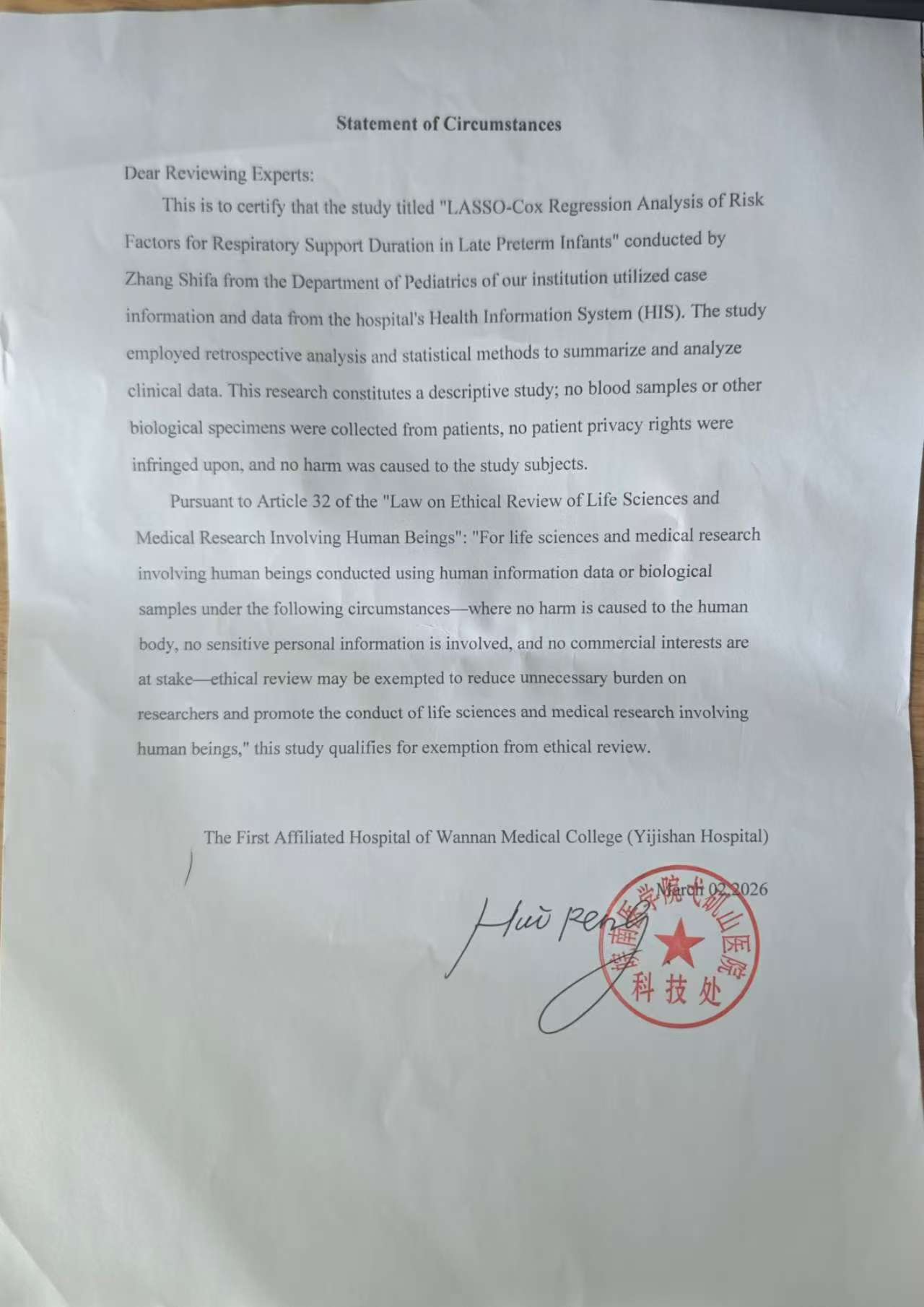

Supplement: Supplementary file 4 [file Image1.jpeg]
